# "Serum and urinary angiotensinogen levels as prognostic indicators in acute kidney injury: a prospective study"

**DOI:** 10.1590/1806-9282.20240971

**Published:** 2024-11-11

**Authors:** Cheng Chen, Jiangtao Zhu

**Affiliations:** 1 Yangzhou Polytechnic College, Department of Medical Science – Yangzhou, China.; 2 Shanxi Provincial Integrated TCM and WM Hospital, Department of Nephrology – Taiyuan, China.

Dear Editor,

We read with interest the article by Akin et al., who found that urinary angiotensinogen levels (corrected by urinary creatinine) can serve as an indicator of the severity of acute kidney injury (AKI)^1^. It is well known that AKI is a common and serious complication in hospitalized patients. Kellum et al. reported that AKI may develop in 18% of general hospitalized patients and half of ICU patients^2^. Currently, the diagnosis of AKI still relies on elevated serum creatinine levels, but its sensitivity is relatively poor. This study found that urinary angiotensinogen level has a high value in the prognosis and management of AKI patients. We congratulate the authors for their valuable contributions, but we still raise some concerns that should be discussed.

First, this study lacks a group of healthy controls. Although the study included 79 AKI patients at different stages, the data are still insufficient, because the expression of angiotensinogen in healthy controls is unclear. Specifically, it is not known whether there is a difference between angiotensinogen levels between healthy individuals and AKI patients in the early stages. Therefore, it is recommended to include a group of healthy controls to further elucidate the trends in angiotensinogen changes.

Second, in this study, the primary cause of AKI was cerebrovascular damage, accounting for 25% of cases. However, the most common cause of AKI is sepsis, followed by major surgery^3^. There is a close relationship between the renin-angiotensin-aldosterone system (RAAS) and cerebrovascular diseases, with RAAS being activated in patients with cerebrovascular disease^4^. This may lead to bias. It is recommended to include more AKI patients induced by sepsis and major surgery.

Third, according to the KDIGO AKI staging, the severity of AKI increases with a higher stage. However, in this study, the authors found that the uAGT/uCr ratio was significantly lower in AKI stage 2 (5.03±5.04) compared to AKI stage 1 (9.93±10.82), but it was significantly higher in AKI stage 3 (32.15±37.13) compared to AKI stage 2 (5.03±5.04). Could this be related to the fact that urine output in AKI stage 3 is significantly reduced compared to stages 1 and 2? If the authors could elaborate on this issue in the discussion, it would make the study more rigorous.
